# Evaluating the Knowledge of and Behavior Toward COVID-19 and the Possibility of Isolating at a City Level: Survey Study

**DOI:** 10.2196/47170

**Published:** 2024-04-11

**Authors:** Elise Verot, Robin Chaux, Julie Gagnaire, Paul Bonjean, Amandine Gagneux-Brunon, Philippe Berthelot, Carole Pelissier, Billal Boulamail, Franck Chauvin, Bruno Pozzetto, Elisabeth Botelho-Nevers

**Affiliations:** 1 CIC EC 1408 INSERM Saint-Etienne Saint-Etienne cedex 2 France; 2 Laboratoire Parcours Santé Systémique- UR4129 Université Jean Monnet Université de Lyon St Priest-en-Jarez France; 3 Chaire Hygée, Institut PRESAGE Université Jean Monnet Université de Lyon Saint-Etienne France; 4 Unité de Recherche Clinique Centre Hospitalier Universitaire de Saint-Etienne Saint-Etienne France; 5 Unité de Gestion des Risques infectieux Centre Hospitalier Universitaire (CHU) de Saint-Etienne Saint-Etienne France; 6 Service d’Infectiologie Centre Hospitalier Universitaire de Saint-Etienne Saint-Etienne France; 7 Team GIMAP, CIRI—Centre International de Recherche en Infectiologie Université Jean Monnet Université de Lyon Saint-Etienne France; 8 Chaire PreVacCI, Institut PRESAGE Université Jean Monnet Université de Lyon Saint-Etienne France; 9 Laboratoire des Agents Infectieux et d’Hygiène Centre Hospitalier Universitaire de Saint-Etienne Saint-Etienne France; 10 Occupational Health Service University Hospital Center of Saint-Etienne Saint-Etienne France; 11 UMRESTTE, Université Lyon 1 Université Gustave Eiffel—IFSTTAR, UMR t 9405 Lyon France

**Keywords:** SARS-CoV-2, COVID-19, health literacy, knowledge, attitude, and perception/practices (KAP), public health, population, mass testing, screening, pandemic, sociological trends, COVID-19 screening

## Abstract

**Background:**

Mass testing campaigns were proposed in France during the first wave of the COVID-19 pandemic to detect and isolate asymptomatic individuals infected by SARS-CoV-2. During mass testing in Saint-Étienne (February 2021), we performed a survey of the general population.

**Objective:**

We evaluated, on the scale of a city’s population, the literacy level about SARS-CoV-2 transmission, barrier gesture respect, and isolation acceptability or possibility in case of SARS-CoV-2 infection.

**Methods:**

We used the validated CovQuest-CC questionnaire. Data were analyzed and correlated with volunteer characteristics and their SARS-CoV-2 screening results using multivariate analysis.

**Results:**

In total, 4707 participants completed the CovQuest-CC questionnaire. Multivariate analysis revealed that female sex was a determinant of a higher score of knowledge about SARS-CoV-2 transmission (adjusted β coefficient=0.14, 95% CI 0.04-0.23; corrected *P*=.02). Older ages of 50-59 years (adjusted β coefficient=0.25, 95% CI 0.19-0.31; corrected *P*<.001) and ≥60 years (adjusted β coefficient=0.25, 95% CI 0.15-0.34; corrected *P*<.001) were determinants of a higher score on barrier gesture respect compared to ages 20-49 years considered as reference. Female sex was also a determinant of a higher score on barrier gesture respect (adjusted β coefficient=0.10, 95% CI 0.02-4.63; corrected *P*<.001). The knowledge score was correlated with the score on barrier gesture respect measures (adjusted β coefficient=0.03, 95% CI 0.001-0.004; corrected *P*=.001). Older ages of 50-59 years (adjusted β coefficient=0.21, 95% CI 0.13-0.29; corrected *P*<.001) and ≥60 years (adjusted β coefficient=0.25, 95% CI 0.1-0.38; corrected *P*<.001) were determinants of a higher score on isolation acceptability or possibility compared to the age of 20-49 years considered as reference. Finally, the knowledge score regarding SARS-CoV-2 transmission was significantly associated with a lower risk of RT-PCR (reverse transcriptase–polymerase chain reaction) positivity (adjusted odds ratio 0.80, 95% CI 0.69-0.94; corrected *P*<.03), implying that a 1-point increase in the knowledge score lowers the risk of positivity by 20% on average.

**Conclusions:**

This study identified factors associated with health literacy regarding SARS-CoV-2 infection in asymptomatic individuals in a large French city’s population. We can confirm that in the context of the COVID-19 pandemic, the determinants of better health literacy are not the same as those in other contexts. It seems critical to obtain a more detailed understanding of the determinants of individual citizens’ behavior, as part of a strategy to combat the large-scale spread of the virus. The harsh experience of this pandemic should teach us how to nurture research to structure customized interventions to encourage the adoption of ad hoc behaviors to engage citizens in adapting behaviors more favorable to their health.

## Introduction

Since the beginning of the COVID-19 crisis, and independent of the wave of the pandemic, one of the main goals of scientists and governments has been to reduce the burden of SARS-CoV-2 infection [1].

### Mass Testing Campaign to Contain the Spread of SARS-CoV-2

Among the different strategies used worldwide, mass testing was proposed early [2], with the aim to detect highly infectious individuals who are asymptomatic or presymptomatic, allowing their isolation together with the rapid identification and testing of their close contacts to reduce virus spread [3]. The performance of this strategy is influenced by the prevalence of active infection in the group being tested. Mass testing was then carried out in high-incidence settings with the hope of mitigating the transmission dynamics and avoiding lockdown measures [2]. The impact of such mass testing was considered controversial [2]. Indeed, although the “test, trace, and isolate” strategy has proven effective in suppressing early spread of SARS-CoV-2 [4,5], this strategy has also shown serious limitations as it has been overwhelmed by the increasing number of cases [5] and associated costs [6]. However, if these interventions were used notably before the availability of safe and effective COVID-19 vaccines, with the emergence of variants of concern exhibiting a high level of transmissibility, testing and isolation-based strategies are likely to remain viable tools for the control of epidemic waves.

### The Citizen as a Player in the Fight Against the Spread of the Virus

Beyond mass nonpharmaceutical interventions conducted by cities or governments [7], each citizen has also a role, and may be an actor, in controlling the epidemics [8-11]. Indeed, a good understanding and knowledge of the transmission route of SARS-CoV-2, adhesion to barrier gesture respect measures, and having realistic possibilities of individual isolation contribute significantly to fight against the pandemic [10-12]. Moreover, after the launch of mass testing or even after a negative test result is obtained, efforts should continue to further protect oneself and others [13]. As West et al [14] pointed out in May 2020, effective interventions are urgently needed to increase adherence of the general population to the proper implementation of health measures for protecting people individually and collectively.

### The Need to Understand Individual Behavior to Tackle the Crisis More Effectively

As Rodon et al [15] point out, assessing health literacy about COVID-19 is crucial to understanding any difficulties individuals may have in adopting protective measures and implementing social distancing measures, bearing in mind that age, education, and the way in which they gather information about COVID-19 are all factors that determine individual behavior. As part of the French health policy in 2021, the city of Saint-Étienne, France (174,082 residents) [16] took part in a French national experiment of city-wide mass testing operations for COVID-19. The campaign took place over 2 full weeks separated by a 5-week interval; the first one occurred from January 13 to 19, markedly after the end-of-year holidays, and the second one occurred from February 22 to 28, when the winter school vacations were over. The city of Saint-Étienne was chosen to be part of this experiment of mass testing since it had high incidence rates, peaking at 1000 cases per 100,000 inhabitants in late 2020 [17,18]. At the time, having been amid this unprecedent crisis, we seized the opportunity of this mass testing operation to survey people about their attitudes and beliefs regarding SARS-CoV-2, which was not undertaken on a city-wide scale. Hence, to our knowledge, this type of evaluation in such a mass testing context among asymptomatic citizens was not performed. While collecting these data may seem outdated at present, given that COVID-19 is no longer a public health emergency of international concern, this large-scale work carried out in a particular situation has enabled us to assess knowledge of, behaviors toward, and opportunities for COVID-19 isolation to better understand our fellow citizens in a pandemic context. At that time, the results of this approach were intended to help develop public health strategies to contain local epidemics using a population approach.

## Methods

The study took place during the second week of the mass testing campaign of Saint-Étienne, France, that is, from February 22 to 28, 2021.

### Background Concerning the Mass Testing Campaign in Saint-Étienne

Adults and children older than 10 years were eligible for inclusion. People wishing to participate in the study had to be older than 10 years and able to read and understand the French language. For minors, parental permission was required. An information note was provided to each participant. After obtaining verbal consent of the participant or of his or her guardian, the participant was included in the study. For SARS-CoV-2 screening, saliva samples were obtained from the participants in accordance with the French guidelines of February 2021 on the use of salivary RT-PCR (reverse transcriptase–polymerase chain reaction) tests in the context of large-scale iterative screening in a closed population. Samples were collected and analyzed using quantitative RT-PCR in accordance with the methods described previously [18].

### Recruitment

Individuals who voluntarily participated in the COVID-19 city-scale mass screening campaign were also offered to complete the CovQuest-CC questionnaire. Screenings were offered free of charge at 12 ephemeral sites, and in parallel, mobile teams were deployed to target populations (adolescents, students, people living in low-income neighborhoods, businesses, etc). Study participants were provided a paper questionnaire with a pen and asked to complete it while they waited to be tested for the presence or absence of SARS-CoV-2. Everyone then handed in the questionnaire at the time of the screening test. The self-administered CovQuest-CC questionnaire explores 3 main areas: knowledge of SARS-CoV-2, adherence to barrier gesture respect, and the ability to isolate themselves in the event of a SARS-CoV-2 infection. For these 3 concept areas, all response modalities are offered in the form of a Likert scale. The precise content of the CovQuest-CC questionnaire is presented elsewhere [19]. The questionnaire was psychometrically tested and validated in a general population aged 10 years and older. The following age groups were represented: 10-19, 20-29, 30-39, 40-49, 50-59, 60-69, 70-79, 80-89, and >90 years. As explained in a previous report on the CovQuest-CC questionnaire validation process [19], we first carried out a pretest with a representative sample of the target population, to assess the comprehensibility of the questions and their wording. [19] The psychometric validation procedure showed this questionnaire was valid, consistent and reliable. Therefore, our study participants were those who both completed the CovQuest-CC questionnaire and were screened for SARS-CoV-2 infection in the context of the mass screening campaign.

### Information Collected From Participants

Participants provided information on demographic characteristics, occupation, place of living, and symptoms through a paper questionnaire. The CovQuest-CC questionnaire provided data through a global knowledge score on SARS-CoV-2 transmission ranging from 0 to 6, a score on barrier gesture respect ranging from 0 to 4, and a score on isolation acceptability or possibility ranging from 0 to 4. The highest score for each item corresponded to the best level of response. The French version of the European Deprivation Index (EDI) was also used in this study to approximate the social deprivation level of participants [20]. This score was calculated in 2007 using homogenous clusters for statistical information (called “IRIS” [regrouped statistical information blocks], originally constructed by the organization in charge of the French census) [21]. EDI quintiles were used to group IRIS into 5 deprivation levels [21]. In this study, participants were associated with a deprivation level (increasing from 1 to 5, with 5 being the most deprived) according to the IRIS area they lived in.

### Ethical Considerations

The CROSS (Checklist for Reporting of Survey Studies) guided the reporting of this survey study [22]. No financial compensation was provided to participants. This study was conducted in accordance with the guidelines set out in the Declaration of Helsinki. The research in which this validation took place was approved by the Ethics Committee of IRB ILE-DE-FRANCE 1 (I ORG0009918; Protocole No. EudraCT: 2021-A00390-41) and all participants provided their written consent. This study is registered on ClinicalTrials.gov (NCT04859023).

### Statistical Analysis

#### Power

The margin of error for our sample of 4707 individuals is 1.45% and corresponds to what is expected for survey studies [23], with a recommended margin of error of 1%-10%.

Sample characteristics were described using frequencies and proportions for categorical variables, and mean, SD, and median and IQR for numerical variables.

The relationship between CovQuest-CC questionnaire scores and variables of interest were analyzed using multivariate linear regression models. The models were constructed a priori, without knowledge of the data. One model was developed to analyze the associations between the knowledge score and age, sex, profession, and EDI quintiles. One model was developed to analyze the associations between the barrier gesture respect score and age, sex, profession, EDI quintiles, and the knowledge score. One model was developed to analyze the associations between the isolation possibility score and age, sex, profession, EDI quintiles, and the number of children at home (one or more versus none). The results are presented as multivariate linear β coefficients with their 95% CI. Colinearity between variables was systematically assessed using correlation matrices and calculation of the variance inflation factor. Models’ validity and robustness were systematically assessed via graphical residual analysis.

Relations between RT-PCR positivity and age, sex, occupation, global SARS-CoV-2 transmission knowledge score, barrier gesture respect score, number of children at home, and EDI were assessed using a multivariate logistic regression model. The results are presented as odds ratios with their 95% CI. To account for multiple testing, Bonferroni correction was used for adjusting the *P* value to the number of variables tested in each model. All statistical tests were 2-sided, with *P*<.05 considered significant. Statistical analysis was performed with the R software (version 4.0.3; The R Foundation).

#### Data Exclusion

Interviewees with at least 1 missing value in one of the variables of the different models were excluded from the said model.

## Results

### User Statistics

Among the 7020 participants in the mass testing campaign, 4707 (67%) responded to the CovQuest-CC questionnaire and were therefore included in this study. The participants’ mean age was 50.3 (SD 18.54) years and their median age was 52 (IQR 36-66) years. Women represented 56.1% (n=2634) of the sample. Among participants, 72.2% (n=3399) lived in the city of Saint-Étienne, and 97.0% (n=4566) of them lived in the Loire or the adjacent Haute-Loire departments; 65.3% (n=2485) of participants lived in areas belonging to the 2 most disadvantaged quintiles of EDI (EDI 4 and EDI 5). Table 1 presents an overview of the sample characteristics.

**Table 1:** Characteristics of study participants (N=4707).

Variable		Value
**Age (years), n (%)**		
	10-19	272 (5.78)
	20-49	1884 (40.06)
	50-59	810 (17.22)
	≥60	1737 (36.93)
	Missing data	4 (0.09)
**Gender, n (%)**		
	Female	2634 (56.14)
	Missing data	15 (0.32)
**Number of children in the household, n (%)**		
	None	3042 (67.42)
	1 or more	1470 (32.58)
	Missing data	195 (4.14)
**Occupation, n (%)**		
	Employees	1820 (38.91)
	Students	370 (7.92)
	High school or college students	152 (3.25)
	Retired	1474 (31.54)
	Unemployed	234 (5.01)
	Health workers	228 (4.88)
	Self-employed workers	142 (3.04)
	Others	254 (5.43)
	Missing data	33 (0.7)
**EDI^a^ quintile, n (%)**		
	First quintile	807 (21.21)
	Second quintile	396 (10.41)
	Third quintile	117 (3.07)
	Fourth quintile	374 (9.83)
	Fifth quintile	2111 (55.48)
	Missing data	902 (19.16)
**SARS-CoV-2 RT-PCR^b^ result, n (%)**		
	Negative	3906 (98.14)
	Positive	74 (1.86)
	Missing data	727 (15.45)
Knowledge score^c^, median (IQR)		5 (4.5-6)
Barrier gesture respect score^d^, median (IQR)		3 (2.50-3.50)
Isolation respect score^e^, median (IQR)		2.75 (2.25-3.33)

^a^EDI: The French version of the European Deprivation Index.

^b^RT-PCR: reverse transcriptase–polymerase chain reaction.

^c^Data missing for 204 (4.33%) participants.

^d^Data missing for 266 (5.65%) participants.

^e^Data missing for 389 (8.26%) participants.

### Evaluation Outcomes

#### Knowledge Score on the Transmission of SARS-CoV-2 According to the CovQuest-CC Questionnaire

**Figure 1:**
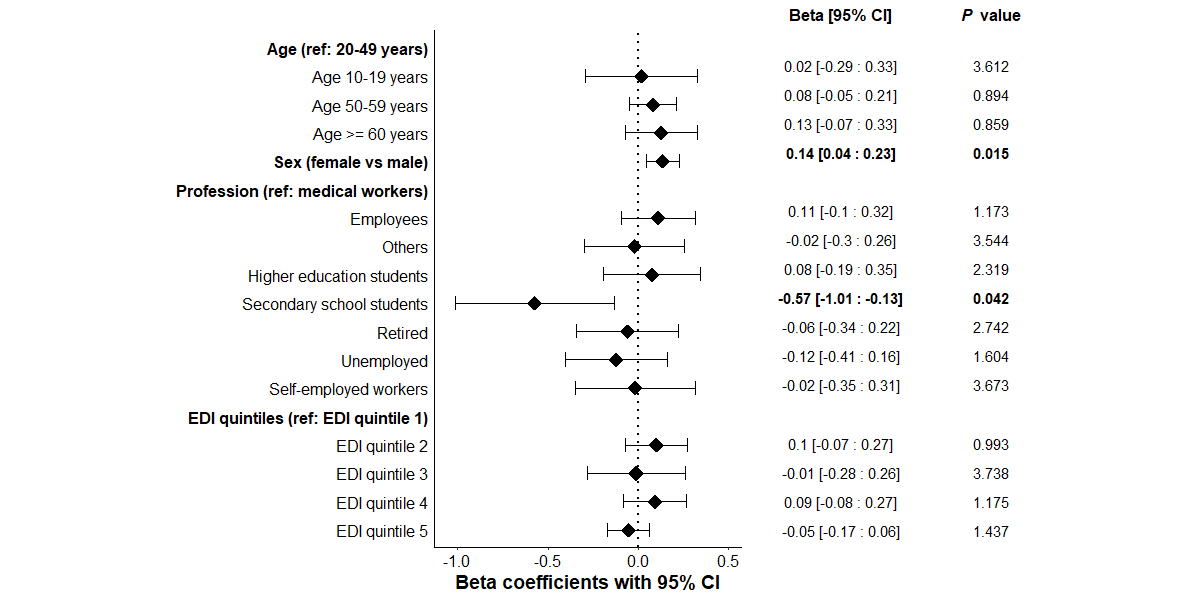
Adjusted multivariate linear regression exploring the associations between the knowledge global score and the variables of interest, with Bonferroni corrected P.

The median score was 5 (IQR 4.5-6). Factors significantly associated with the knowledge score in multivariate analysis are depicted in Figure 1 and Multimedia Appendix 1. Female sex was shown as a determinant of a higher score for knowledge about transmission of SARS-CoV-2 (adjusted β coefficient=0.14, 95% CI 0.04-0.23; corrected *P*=.02). Compared to health workers taken as reference, middle and high school students exhibited a lower score of knowledge about transmission of SARS-CoV-2 (adjusted β coefficient=–0.57, 95% CI –1.01 to –0.13; corrected *P*=.04).

#### Barrier Gesture Respect Measure Scores According to the CovQuest-CC Questionnaire

**Figure 2:**
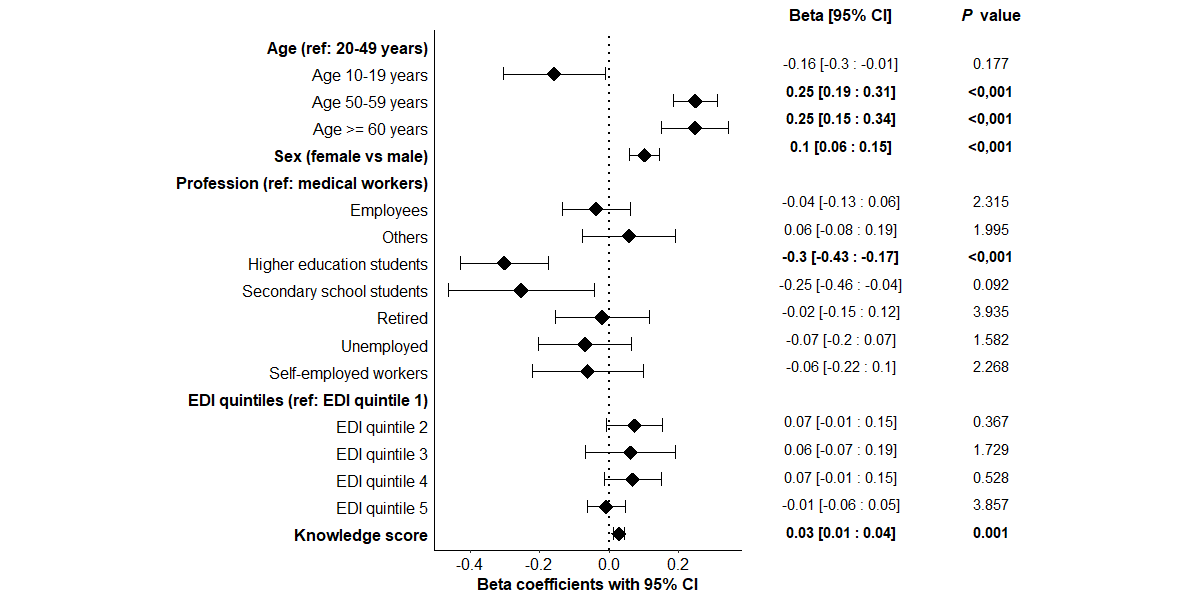
Adjusted multivariate linear regression exploring the associations between the barrier gesture respect score and the variables of interest, with Bonferroni corrected P.

The median score was 3 (IQR 2.5-3.5). Factors significantly associated with this score in multivariate analysis are depicted in Figure 2 and Multimedia Appendix 2. Older ages of 50-59 years (adjusted β coefficient=0.25, 95% CI 0.19-0.31; corrected *P*<.001) and ≥60 years (adjusted β coefficient=0.25, 95% CI 0.15-0.34; corrected *P*<.001) were shown as determinants of a higher score on barrier gesture respect compared to ages 20-49 years considered as reference. Female sex also appeared as a determinant of a higher score on barrier gesture respect (adjusted β coefficient=0.10, 95% CI 0.02-4.63; corrected *P*<.001). Finally, the level of the knowledge score was also correlated to that of the score for barrier gesture respect measures (adjusted β coefficient=0.03, 95% CI 0.001-0.004; corrected *P*=.001). By contrast, higher education was significantly associated with a lower barrier gesture respect score (adjusted β coefficient=–0.30, 95% CI –0.43 to –0.17; corrected *P*<.001).

#### Score for Isolation Acceptability or Possibility According to the CovQuest-CC Questionnaire

**Figure 3:**
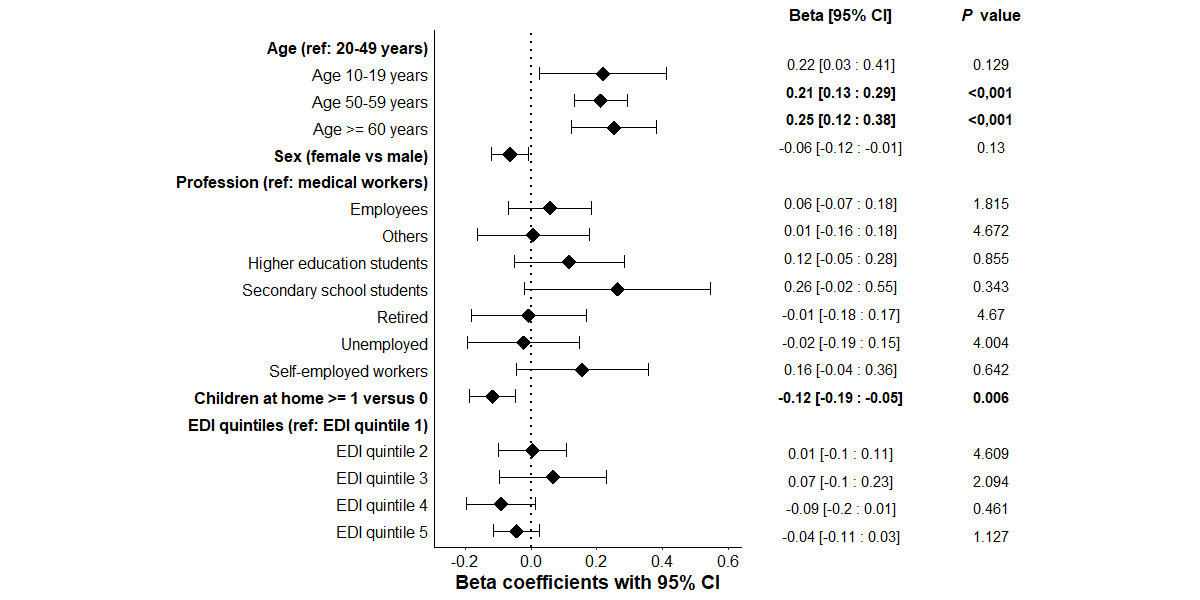
Adjusted multivariate linear regression exploring the associations between the isolation possibility score and the variables of interest, with Bonferroni corrected P.

The median score was 2.75 (IQR 2.25-3.33). Factors significantly associated with this score in multivariate analysis are depicted in Figure 3 and Multimedia Appendix 3. Older ages of 50-59 years (adjusted β coefficient=0.21, 95% CI 0.13-0.29; corrected *P*<.001) and ≥60 years (adjusted β coefficient=0.25, 95% CI 0.1-0.38; corrected *P*<.001) were shown to be determinants of a higher score on isolation acceptability or possibility compared to ages 20-49 years considered as reference. In contrast, having at least 1 child living at home versus having none exhibited a lower score on isolation possibility (adjusted β coefficient=–0.12, 95% CI –0.19 to –0.05; corrected *P*=.006).

#### RT-PCR Positivity Determinants

**Figure 4:**
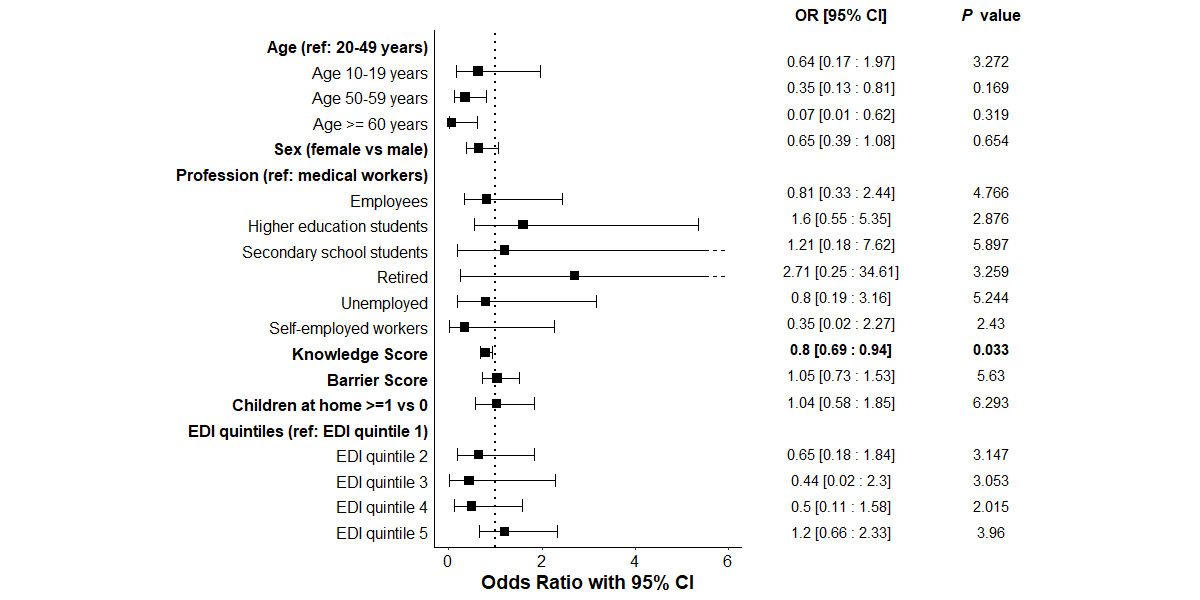
Adjusted multivariate logistic regression exploring the associations between PCR positivity and the variables of interest, with Bonferroni corrected P.

Results of univariate and multivariate analyses of RT-PCR positivity determinants are presented in Figure 4 and Multimedia Appendix 4. Interestingly, the knowledge score for SARS-CoV-2 transmission was significantly associated with a lower risk of RT-PCR positivity (adjusted odds ratio 0.80, 95% CI 0.69-0.94; corrected *P*<.03), implying that a 1-point increase in knowledge score lowers the risk of positivity by 20% on average.

## Discussion

### Background

In the middle of a city-wide mass screening campaign, beyond proposing massive SARS-CoV-2 screening for asymptomatic citizens, as accomplished in several other cities [24,25], we were able to assess the knowledge of, behavior toward, and potential for isolation of participants through a validated questionnaire on SARS-CoV-2–related health literacy [19]. It therefore provided a global picture of the determinants of the population’s adherence to a national control strategy in the context of a pandemic at the level of a French semiurban agglomeration of 174,082 inhabitants.

Indeed, the sanitary crisis that has lasted for 3 years now, has been associated with the necessary implementation of social and individual constraints that have not always been accepted or adopted by the general population despite their crucial role to curb the epidemic, reflecting a lack of health literacy in the general population [12]. These deficiencies were further reinforced by discordant messages issued from medical and nonmedical influencers or by deviation of control strategies that were misunderstood because of their complexity [12]. As Hannah Spring pointed out in an editorial on the COVID-19 pandemic, “good health literacy has never been more crucial for survival” [12].

### Principal Results

Our results show that female sex was an independent determinant of better knowledge of SARS-CoV-2. We were also able to find evidence that middle and high school status was related to a low score of knowledge about SARS-CoV-2. Same gender and age distribution were also associated with a better knowledge score in a recent study conducted by our Belgian neighbors [26]. Better health literacy among older people seems, however, to be specific to the COVID-19 pandemic, as previous studies have shown that older age is a determinant of lower health literacy [27-30].

According to our findings, the social gradient did not appear as a determinant of better health literacy regarding COVID-19. The same observation was reported by Okan et al [31] in the German population. They suggested that the lack of a social gradient may mean that a great deal of information about the COVID-19 epidemic and SARS-CoV-2 has been made available and understandable, and that the informational environment helps to build health literacy on the topic [31]. It contrasts with other contexts than the COVID-19 pandemic, where better health literacy is usually observed in higher social classes [32]. Our results emphasize that the EDI cannot be settled as a determinant of health literacy, SARS-CoV-2 positivity, or compliance with existing health measures. During the first wave of the epidemic, it appeared that French and Italian citizens—2 populations generally known for their tolerance of deviation from sanitary norms—appeared to be much more disciplined and more respectful of the recommendations issued by public health authorities than Chinese, German, or British citizens, which can be considered as paradoxical [33,34]. Indeed, several studies have suggested that preventive health behavior is strongly influenced by social norms [33,35,36]. We can then hypothesize that, in France, during the beginning of the pandemic, citizens became compliant to sanitary rules as a new normative reference [37].

Factors independently associated with a higher respect of barrier gestures were older age and female sex. Of note, we also found a positive correlation with global knowledge scores. In a recent study on the determinants of preventive behaviors in response to the COVID-19 pandemic in France, we showed that men and young adults were less likely to follow guidelines to contain the spread of COVID-19 [33]. Our results are consistent with this in terms of the age and gender distribution in this study. Other international studies highlighted that better health literacy during the pandemic was associated with adherence to preventive behaviors, which is in line with our results [38].

We also had the opportunity to evaluate willingness and the possibility of isolation in case of SARS-CoV-2 infection. Isolation of COVID-19–positive individuals is crucial to contain an epidemic and is a factor that, to our knowledge, has not been well studied before. Our results show that determinants of a higher self-isolation possibility are older age and having no children living under the same roof. Previous literature suggests that socioeconomic status and a fear of loss of income due to home quarantine are the main barriers for adhering to self-isolation [39-41]. It is particularly true among the lowest socioeconomic status quintiles where people are usually unable to work from home [41]. During the pilot study of asymptomatic testing in Liverpool, the United Kingdom, one of the main barriers to adherence to self-isolation was the fear of testing positive and not having sufficient support to implement this individual measure or experiencing a loss of income [42]. In France, home quarantine was not associated with loss of income as the government compensated the loss of salary [43]. In our study, we showed that the major obstacle to the effective implementation of self-isolation was housing conditions, especially with households having more than 1 child. Once again, the EDI was not independently associated with isolation. Self-discipline is, however, not enough; the right conditions must be in place to minimize the risk of disrupting family or social dynamics and thus to produce the expected effects. Practical dispositions such as living conditions, ability to access basic supplies and needs, and access to health care are important factors in individuals’ decisions to comply or not with self-isolation and also possibly participate in a screening campaign [40].

Regarding RT-PCR positivity, it was interesting to observe that a significant determinant of a SARS-CoV-2 infection was the knowledge score regarding SARS-CoV-2 transmission. A 2021 report from the Norwegian Institute of Public Health showed that people with low education and low family income have higher rates of confirmed positive cases [44]. Nevertheless, we cannot make the same claim for our population, given that we did not record the school education level of our respondents. Other studies have confirmed the existence of sociodemographic factors associated with COVID-19 such as deprivation [45]. In our study, belonging to deprived EDI strata or to a low socio-professional category was not independently associated with SARS-CoV-2 infection. However, detection of SARS-CoV-2 infection in the study participants was performed only on 1 day, which was not the case in other studies.

As Schmidt et al [38] recently reported, one of the key elements to be retained from the various recent studies carried out is that the level of health literacy specific to COVID-19 seems to be a strong determinant of the individual’s commitment to adopting preventive attitudes and behaviors toward the virus [38].

### Limitations

Our study has some limitations. First, Saint-Étienne ranks 14th among France’s 75 most populous cities, and 17th among 50 of France’s main urban areas. So, despite its position in the top half of France’s most populous cities, it is by no means representative of all French cities [16,46].

Second, we used EDI and IRIS to determine socioeconomic status and geographic areas. We were, therefore, unable to classify individuals by rural, periurban, or urban catchment area since EDI was created in 2007 and the urban landscape has been reshaped. Thus, it was not possible for us to verify whether there is a difference in the level of health literacy according to urban or rural areas. However, it is important to note that the literature is mixed on this specific issue [47-51]. Moreover, EDI is an area indicator and its transposition at an individual level may not be representative of the true deprivation status in numerous cases. Recording the socioeconomic status of individuals would have been more precise but would have increased the length of the questionnaire and may have stigmatized people; hence, we did not make this choice.

We excluded participants with missing values, which can limit the representativeness of the study sample relative to the target population. The most frequent reason for exclusion was the impossibility to determine the IRIS area where participants lived. A proportion of exclusions was also due to incomplete filling of the questionnaire, resulting in an impossibility to calculate scores for 4% to 8% of participants, depending on the score. We cannot rule out a bias of social desirability in the declarative responses to questions about respondents’ behavior with respect to their adherence to existing barrier practices. Moreover, we cannot exclude a representation bias as volunteer participants self-presented to take part in a mass screening campaign and were probably already aware of strategies deployed to contain the epidemic, and their behaviors were perhaps not representative of those of the general population. Especially since the demographic characteristics of our population are slightly different from those of the French general population, we had a notably higher proportion of older individuals [52]. However, we analyzed their behaviors according to their sociodemographic characteristics. Nevertheless, the context, the population surveyed led us to acknowledge that the results of this study have limited generalization. Furthermore, the results obtained were not used to implement public health interventions since the epidemic situation improved. However, our results could help customize specific avenues in case of a future emergency.

### Comparison With Prior Work

#### Added Value of This Study

To our knowledge, this is the first study to assess the knowledge of, behavior toward, and isolation for COVID-19 in a large-scale population during a mass city-wide testing using a validated questionnaire (CovQuest-CC questionnaire) [19]. This evaluation was correlated with the results of the SARS-CoV-2 RT-PCR testing performed for each individual during the mass city-wide testing. A correlation was found between knowledge scores and positive findings on RT-PCR testing among asymptomatic people. In the setting of the COVID-19 pandemic, determinants of better health literacy were not the same as those in other contexts. Other works have evaluated such types of data on a large scale but never in the context of city-wide mass testing and with a lower number of respondents [26,31,33].

#### Implications of all Available Evidence

As pointed out by Mühlbacher et al [53], restrictive measures in the event of a pandemic can only be successful if they are accepted by the population and if political decision makers can count on the approval of a large with the majority of citizens.

In this way, knowledge of behavioral determinants can help to implement appropriate nonpharmaceutical interventions, supported by public commitment, in the event of a large-scale health threat.

Thus, assessing health-related knowledge about SARS-CoV-2 is crucial, particularly in a large population, in order to transition to public health intervention research, which will provide essential multilevel responses at organizational, societal, and individual levels, while enabling the implementation of structured, individualized interventions aimed at different typologies of individuals.

### Conclusions

In conclusion, this study identifies factors associated with health literacy regarding SARS-CoV-2 infection in asymptomatic individuals in a large French population. We can confirm that, in the context of the COVID-19 pandemic, the determinants of better health literacy are not the same as those in other contexts. In particular, we were able to establish a significant relationship between a low health literacy score on SARS-CoV-2 and positivity in an asymptomatic population. We were also able to highlight that female gender is an independent determinant of a better level of health literacy regarding SARS-CoV-2, and that middle and high school status signaled a low level. It seems extremely important to obtain a more detailed understanding of the determinants of individual citizens’ behavior as part of a strategy to combat the large-scale spread of a virus. The harsh experience of this pandemic should teach us how to nurture research to structure customizable interventions to encourage the adoption of ad hoc behaviors to engage citizens in adapting behaviors more favorable to their health.

## Appendix

Multimedia Appendix 1 Detailed univariate and adjusted multivariate linear regression exploring the associations between the Knowledge Global Score and the variables of interest, with non-corrected P and Bonferroni corrected P for the multivariate analysis.

Multimedia Appendix 2 Detailed univariate and adjusted multivariate linear regression exploring the associations between the Barrier Gesture Respect Score and the variables of interest, with non-corrected P and Bonferroni corrected P for the multivariate analysis.

Multimedia Appendix 3 Detailed univariate and adjusted multivariate linear regression exploring the associations between the Isolation Possibility Score and the variables of interest, with non-corrected P and Bonferroni corrected P for the multivariate analysis.

Multimedia Appendix 4 Detailed univariate and adjusted multivariate logistic regression exploring the associations between PCR positivity and the variables of interest, with non-corrected P and Bonferroni corrected P for the multivariate analysis.

## Abbreviations

CROSS: Checklist for Reporting of Survey Studies
EDI: European Deprivation Index
IRIS: regrouped statistical information blocks
RT-PCR: reverse transcriptase–polymerase chain reaction

## Data Availability

The data sets generated during or analyzed during this study are available from the corresponding author on reasonable request.
