# Supplementary material for: Evaluating the Knowledge of and Behavior Toward COVID-19 and the Possibility of Isolating at a City Level: Survey Study
Source: JMIR Public Health Surveill. 2024 Apr 11;10:e47170. doi: 10.2196/47170 (PMC11013031; doi:10.2196/47170)
Supplement: Multimedia Appendix 1 [file publichealth_v10i1e47170_app1.docx]

**Supplementary table 1 –** Detailed univariate and adjusted multivariate linear regression exploring the associations between the Knowledge Global Score and the variables of interest, with non-corrected *P* and Bonferroni corrected *P* for the multivariate analysis*.*

| **Variables** | | **Knowledge score** | **Univariate Analysis** | **Multivariate Analysis*** | | |
| --- | --- | --- | --- | --- | --- | --- |
|  |  | **Mean (± sd)** | **β [95% CI]** | **β [95% CI]** | ***p-value*** | ***corrected p-value***** |
| **Age** | |  |  |  |  |  |
|  | *Ref= 20-49 years* | 4.77 (± 1.37) | - | - | - | - |
|  | 10 - 19 years | 4.48 (± 1.44) | -0.29 [-0.47 : -0.11] | 0.02 [-0.29 : 0.33] | 0.903 | 1 |
|  | 50 - 59 years | 4.82 (± 1.37) | 0.05 [-0.07 : 0.16] | 0.08 [-0.05 : 0.21] | 0.224 | 0.894 |
|  | >= 60 years | 4.78 (± 1.41) | 0.01 [-0.09 : 0.10] | 0.13 [-0.07 : 0.33] | 0.215 | 0.859 |
| **Gender** | |  |  |  |  |  |
|  | *Ref= Male* | 4.7 (± 1.44) | - | - | - | - |
|  | Female | 4.82 (± 1.35) | 0.12 [0.04 : 0.21] | 0.14 [0.04 : 0.23] | **0.004** | **0.015** |
| **Occupation** | |  |  |  |  |  |
|  | *Ref = Health workers* | 4.77 (± 1.35) | - | - | - | - |
|  | Employees | 4.83 (± 1.34) | 0.06 [-0.13 : 0.25] | 0.11 [-0.09 : 0.32] | 0.293 | 1 |
|  | Others | 4.7 (± 1.53) | -0.07 [-0.32 : 0.18] | -0.02 [-0.29 : 0.26] | 0.886 | 1 |
|  | Students | 4.84 (± 1.24) | 0.07 [-0.16 : 0.29] | 0.08 [-0.19 : 0.35] | 0.579 | 1 |
|  | High school, college students | 4.15 (± 1.62) | -0.62 [-0.91 : -0.34] | -0.57 [-1.01 : -0.13] | **0.01** | **0.042** |
|  | Retired | 4.77 (± 1.41) | -0.01 [-0.19 : 0.19] | -0.06 [-0.34 : 0.22] | 0.686 | 1 |
|  | Unemployed | 4.63 (± 1.6) | -0.15 [-0.40 : 0.11] | -0.12 [-0.41 : 0.16] | 0.401 | 1 |
|  | Self employed | 4.72 (± 1.19) | -0.06 [-0.35 : 0.24] | -0.02 [-0.35 : 0.31] | 0.918 | 1 |
| **EDI quintile** | |  |  |  |  |  |
|  | *Ref= quintile 1* | 4.79 (± 1.33) | - | - | - | - |
|  | EDI quintile 2 | 4.88 (± 1.37) | 0.09 [-0.07 : 0.26] | 0.09 [-0.07 : 0.27] | 0.248 | 0.993 |
|  | EDI quintile 3 | 4.77 (± 1.32) | -0.02 [-0.29 : 0.26] | -0.01 [-0.28 : 0.26] | 0.934 | 1 |
|  | EDI quintile 4 | 4.88 (± 1.39) | 0.09 [-0.08 : 0.27] | 0.09 [-0.08 : 0.27] | 0.294 | 1 |
|  | EDI quintile 5 | 4.72 (± 1.41) | -0.07 [-0.18 : 0.05] | -0.05 [-0.17 : 0.06] | 0.359 | 1 |
| ** Variables displayed in the table are the variables included in the multivariate analysis* | | | | |  |  |
| *** Bonferroni corrected p-value* | |  |  |  |  |  |
