# Supplementary material for: Evaluating the Knowledge of and Behavior Toward COVID-19 and the Possibility of Isolating at a City Level: Survey Study
Source: JMIR Public Health Surveill. 2024 Apr 11;10:e47170. doi: 10.2196/47170 (PMC11013031; doi:10.2196/47170)
Supplement: Multimedia Appendix 2 [file publichealth_v10i1e47170_app2.docx]

**Supplementary table 2 –** Detailed univariate and adjusted multivariate linear regression exploring the associations between the Barrier Gesture Respect Score and the variables of interest, with non-corrected *P* and Bonferroni corrected *P* for the multivariate analysis.

| **Variables** | | **Barrier gesture score** | **Univariate Analysis** | **Multivariate Analysis*** | | |
| --- | --- | --- | --- | --- | --- | --- |
|  |  | **Mean (± sd)** | **β [95% CI]** | **β [95% CI]** | ***P*** | ***corrected P***** |
| **Age** |  |  |  |  |  |  |
|  | *Ref= 20-49 years* | 2.82 (± 0.7) | - | - | - | - |
|  | 10 - 19 years | 2.46 (± 0.75) | -0.35 [-0.44 : -0.27] | -0.16 [-0.30 : -0.01] | **0.035** | 0.177 |
|  | 50 - 59 years | 3.09 (± 0.6) | 0.28 [0.22 : 0.33] | 0.25 [0.19 : 0.31] | **<0.001** | **<0.001** |
|  | >= 60 years | 3.1 (± 0.62) | 0.28 [0.24 : 0.32] | 0.25 [0.15 : 0.34] | **<0.001** | **<0.001** |
| **Gender** | |  |  |  |  |  |
|  | *Ref= Male* | 2.89 (± 0.7) | - | - | - | - |
|  | Female | 2.99 (± 0.66) | 0.09 [0.05 : 0.13] | 0.10 [0.06 : 0.15] | **<0.001** | **<0.001** |
| **Occupation** | |  |  |  |  |  |
|  | *Ref = Health workers* | 2.99 (± 0.68) | - | - | - | - |
|  | Employees | 2.95 (± 0.65) | -0.04 [-0.13 : 0.05] | -0.04 [-0.13 : 0.06] | 0.463 | 1 |
|  | Others | 3.03 (± 0.68) | 0.04 [-0.08 : 0.16] | 0.06 [-0.08 : 0.19] | 0.399 | 1 |
|  | Students | 2.51 (± 0.69) | -0.48 [-0.59 : -0.37] | -0.30 [-0.43 : -0.17] | **<0.001** | **<0.001** |
|  | High school, college students | 2.5 (± 0.76) | -0.49 [-0.33 : -0.35] | -0.25 [-0.46 : -0.04] | **0.018** | 0.092 |
|  | Retired | 3.09 (± 0.63) | 0.11 [0.01 : 0.20] | -0.02 [-0.15 : 0.12] | 0.787 | 1 |
|  | Unemployed | 2.88 (± 0.78) | -0.11 [-0.23 : 0.01] | -0.07 [-0.20 : 0.07] | 0.316 | 1 |
|  | Self employed | 2.95 (± 0.72) | -0.04 [-0.18 : 0.10] | -0.06 [-0.22 : 0.09] | 0.454 | 1 |
| **EDI quintile** | |  |  |  |  |  |
|  | *Ref= quintile 1* | 2.94 (± 0.64) | - | - | - | - |
|  | EDI quintile 2 | 3.04 (± 0.65) | 0.10 [0.02 : 0.19] | 0.07 [-0.01 : 0.15] | 0.073 | 0.367 |
|  | EDI quintile 3 | 3 (± 0.59) | 0.06 [-0.07 : 0.19] | 0.06 [-0.07 : 0.19] | 0.346 | 1 |
|  | EDI quintile 4 | 3.04 (± 0.62) | 0.09 [0.01 : 0.18] | 0.07 [-0.01 : 0.15] | 0.106 | 0.528 |
|  | EDI quintile 5 | 2.93 (± 0.71) | -0.01 [-0.06 : 0.05] | -0.01 [-0.06 : 0.05] | 0.771 | 1 |
| **Knwoledge score***** | |  |  |  |  |  |
|  | knowledge score < median | 2.85 (± 0.71) | 0.04 [0.03 : 0.06] | 0.03 [0.01 : 0.04] | **<0.001** | **0.001** |
|  | knowledge score >= median | 3.03 (± 0.64) |  |  |  |  |
| ** Variables displayed in the table are the variables included in the multivariate analysis* | | | | |  |  |
| *** Bonferroni corrected p-value* | |  |  |  |  |  |
| **** Dichotomized at the median for description, used as a quantitative variable for modeling* | | | | |  |  |
