# Supplementary material for: Evaluating the Knowledge of and Behavior Toward COVID-19 and the Possibility of Isolating at a City Level: Survey Study
Source: JMIR Public Health Surveill. 2024 Apr 11;10:e47170. doi: 10.2196/47170 (PMC11013031; doi:10.2196/47170)
Supplement: Multimedia Appendix 3 [file publichealth_v10i1e47170_app3.docx]

**Supplementary table 3 –** Detailed univariate and adjusted multivariate linear regression exploring the associations between the Isolation Possibility Score and the variables of interest, with non-corrected *P* and Bonferroni corrected *P* for the multivariate analysis.

| **Variables** | | **Isolation Score** | **Univariate Analysis** | **Multivariate Analysis*** | | |
| --- | --- | --- | --- | --- | --- | --- |
|  |  | **Mean (± sd)** | **β [95% CI]** | **β [95% CI]** | ***p-value*** | ***corrected P***** |
| **Age** |  |  |  |  |  |  |
|  | *Ref= 20-49 years* | 2.6 (± 0.86) | - | **-** | - | - |
|  | 10 - 19 years | 2.9 (± 0.89) | 0.31 [0.19 : 0.42] | 0.22 [0.03 : 0.41] | 0.026 | 0.129 |
|  | 50 - 59 years | 2.82 (± 0.81) | 0.22 [0.15 : 0.29] | 0.21 [0.13 : 0.29] | <0.001 | **<0.001** |
|  | >= 60 years | 2.85 (± 0.78) | 0.25 [0.20 : 0.31] | 0.25 [0.12 : 0.38] | <0.001 | **<0.001** |
| **Gender** | |  |  |  |  |  |
|  | *Ref= Male* | 2.8 (± 0.8) | - | - | - | **-** |
|  | Female | 2.7 (± 0.86) | -0.09 [-0.15 : -0.05] | -0.06 [-0.12 : -0.01] | 0.026 | 0.129 |
| **Occupation** | |  |  |  |  |  |
|  | *Ref = Health workers* | 2.59 (± 0.88) | - | - | - | - |
|  | Employees | 2.7 (± 0.84) | 0.12 [0.003 : 0.24] | 0.06 [-0.07 : 0.18] | 0.363 | 1 |
|  | Others | 2.68 (± 0.9) | 0.09 [-0.06 : 0.24] | 0.01 [-0.16 : 0.18] | 0.934 | 1 |
|  | Students | 2.71 (± 0.85) | 0.13 [-0.01 : 0.27] | 0.12 [-0.05 : 0.28] | 0.171 | 0.855 |
|  | High school, college students | 2.97 (± 0.9) | 0.39 [0.2 : 0.58] | 0.26 [-0.02 : 0.55] | 0.069 | 0.343 |
|  | Retired | 2.83 (± 0.77) | 0.25 [0.13 : 0.37] | -0.01 [-0.18 : 0.17] | 0.934 | 1 |
|  | Unemployed | 2.61 (± 0.92) | 0.02 [-0.13 : 0.18] | -0.02 [-0.19 : 0.15] | 0.801 | 1 |
|  | Self employed | 2.78 (± 0.78) | 0.19 [0.02 : 0.37] | 0.16 [-0.04 : 0.36] | 0.128 | 0.642 |
| **Number of children in the household** | |  |  |  |  |  |
|  | *Ref = no child in the household* | 2.8 (± 0.81) | - | - | - | - |
|  | 1 or more | 2.62 (± 0.86) | -0.18 [-0.24 : -0.13] | -0.12 [-0.19 : 0.11] | 0.001 | **0.006** |
| **EDI quintile** | |  |  |  |  |  |
|  | *Ref= quintile 1* | 2.77 (± 0.82) | - | - | - | - |
|  | EDI quintile 2 | 2.79 (± 0.83) | 0.02 [-0.09 : 0.12] | 0.01 [-0.09 : 0.11] | 0.922 | 1 |
|  | EDI quintile 3 | 2.87 (± 0.75) | 0.09 [-0.07 : 0.26] | 0.07 [-0.09 : 0.23] | 0.419 | 1 |
|  | EDI quintile 4 | 2.7 (± 0.83) | -0.07 [-0.18 : 0.03] | -0.09 [-0.19 : 0.01] | 0.092 | 0.461 |
|  | EDI quintile 5 | 2.75 (± 0.84) | -0.02 [-0.09 : 0.05] | -0.04 [-0.11 : 0.03] | 0.225 | 1 |
| ** Variables displayed in the table are the variables included in the multivariate analysis* | | | | |  |  |
| *** Bonferroni corrected p-value* | |  |  |  |  |  |
