# Supplementary material for: Evaluating the Knowledge of and Behavior Toward COVID-19 and the Possibility of Isolating at a City Level: Survey Study
Source: JMIR Public Health Surveill. 2024 Apr 11;10:e47170. doi: 10.2196/47170 (PMC11013031; doi:10.2196/47170)
Supplement: Multimedia Appendix 4 [file publichealth_v10i1e47170_app4.docx]

**Supplementary table 4 –** Detailed univariate and adjusted multivariate logistic regression exploring the associations between PCR positivity and the variables of interest, with non-corrected *P* and Bonferroni corrected *P* for the multivariate analysis.

| **Variables** | | **PCR positivity description** | | **Univariate Analysis** | **Multivariate Analysis*** | | |
| --- | --- | --- | --- | --- | --- | --- | --- |
|  |  | **PCR -, n (%)** | **PCR +, n (%)** | **OR [95% CI]** | **OR [95% CI]** | ***p-value*** | ***corrected P***** |
| **Age** |  |  |  |  |  |  |  |
|  | *Ref= 20-49 years* | 1560 (39.94) | 51 (39.94) | - | - | - | - |
|  | 10 - 19 years | 217 (5.56) | 6 (5.56) | 0.85 [0.32 : 1.84] | 0.64 [0.17 : 1.97] | 0.467 | 1 |
|  | 50 - 59 years | 689 (17.64) | 6 (17.64) | 0.27 [0.1 : 0.58] | 0.35 [0.13 : 0.81] | 0.024 | 0.169 |
|  | >= 60 years | 1440 (36.87) | 11 (36.87) | 0.23 [0.12 : 0.43] | 0.07 [0.01 : 0.62] | 0.046 | 0.319 |
| **Gender** | |  |  |  |  |  |  |
|  | *Ref= Male* | 1689 (43.25) | 44 (43.25) |  |  |  |  |
|  | Female | 2216 (56.75) | 30 (56.75) | 0.52 [0.32 : 0.83] | 0.65 [0.39 : 1.08] | 0.093 | 0.654 |
| **Occupation** | |  |  |  |  |  |  |
|  | Health workers | 197 (5.38) | 6 (5.38) | - | - | - | - |
|  | Students | 291 (7.94) | 14 (7.94) | 1.58 [0.62 : 4.52] | 1.6 [0.55 : 5.35] | 0.411 | 1 |
|  | High school, college students | 120 (3.28) | 3 (3.28) | 0.82 [0.17 : 3.17] | 1.21 [0.18 : 7.62] | 0.842 | 1 |
|  | Retired | 1217 (33.22) | 10 (33.22) | 0.27 [0.1 : 0.8] | 2.71 [0.25 : 34.61] | 0.466 | 1 |
|  | Unemployed | 195 (5.32) | 6 (5.32) | 1.01 [0.31 : 3.28] | 0.8 [0.19 : 3.16] | 0.749 | 1 |
|  | Employees | 1529 (41.73) | 31 (41.73) | 0.67 [0.29 : 1.79] | 0.81 [0.33 : 2.44] | 0.681 | 1 |
|  | Self employed | 115 (3.14) | 2 (3.14) | 0.57 [0.08 : 2.53] | 0.35 [0.02 : 2.27] | 0.347 | 1 |
| **Knwoledge score***** | |  |  |  |  |  |  |
|  | knowledge score < median | 1878 (50.17) | 46 (50.17) | 0.82 [0.71 : 0.94] | 0.8 [0.69 : 0.94] | 0.005 | 0.033 |
|  | knowledge score >= median | 1865 (49.83) | 28 (49.83) |  |  |  |  |
| **Barrier gesture score***** | |  |  |  |  |  |  |
|  | Barrier gesture score < median | 1541 (41.75) | 38 (41.75) | 0.71 [0.52 : 0.98] | 1.05 [0.73 : 1.53] | 0.804 | 1 |
|  | Barrier gesture score >= median | 2150 (58.25) | 34 (58.25) |  |  |  |  |
| **Number of children in the household** | |  |  |  |  |  |  |
|  | *Ref = no child in the household* | 2530 (67.52) | 45 (67.52) | 1.29 [0.79 : 2.07] | 1.04 [0.58 : 1.85] | 0.899 | 1 |
|  | 1 or more | 1217 (32.48) | 28 (32.48) |  |  |  |  |
| **EDI quintile** | |  |  |  |  |  |  |
|  | *Ref= quintile 1* | 769 (21.31) | 14 (21.31) | - | - | - | - |
|  | EDI quintile 2 | 378 (10.48) | 6 (10.48) | 0.87 [0.31 : 2.19] | 0.65 [0.18 : 1.84] | 0.45 | 1 |
|  | EDI quintile 3 | 113 (3.13) | 1 (3.13) | 0.49 [0.03 : 2.45] | 0.44 [0.02 : 2.3] | 0.436 | 1 |
|  | EDI quintile 4 | 353 (9.78) | 3 (9.78) | 0.47 [0.11 : 1.44] | 0.5 [0.11 : 1.58] | 0.288 | 1 |
|  | EDI quintile 5 | 1995 (55.29) | 43 (55.29) | 1.18 [0.66 : 2.25] | 1.2 [0.66 : 2.33] | 0.566 | 1 |
| ** Variables displayed in the table are the variables included in the multivariate analysis* | | | | |  |  |  |
| *** Bonferroni corrected P* | |  |  |  |  |  |  |
| **** Dichotomized at the median for description, used as a quantitative variable for modeling* | | | | |  |  |  |
